# Mammalian PRC1 Complexes: Compositional Complexity and Diverse Molecular Mechanisms

**DOI:** 10.3390/ijms21228594

**Published:** 2020-11-14

**Authors:** Zhuangzhuang Geng, Zhonghua Gao

**Affiliations:** 1 Departments of Biochemistry and Molecular Biology, Penn State College of Medicine, Hershey, PA 17033, USA; zgeng@pennstatehealth.psu.edu; 2 Penn State Hershey Cancer Institute, Hershey, PA 17033, USA; 3 The Stem Cell and Regenerative Biology Program, Penn State College of Medicine, Hershey, PA 17033, USA

**Keywords:** PRC1, transcription regulation, chromatin structure

## Abstract

Polycomb group (PcG) proteins function as vital epigenetic regulators in various biological processes, including pluripotency, development, and carcinogenesis. PcG proteins form multicomponent complexes, and two major types of protein complexes have been identified in mammals to date, Polycomb Repressive Complexes 1 and 2 (PRC1 and PRC2). The PRC1 complexes are composed in a hierarchical manner in which the catalytic core, RING1A/B, exclusively interacts with one of six Polycomb group RING finger (PCGF) proteins. This association with specific PCGF proteins allows for PRC1 to be subdivided into six distinct groups, each with their own unique modes of action arising from the distinct set of associated proteins. Historically, PRC1 was considered to be a transcription repressor that deposited monoubiquitylation of histone H2A at lysine 119 (H2AK119ub1) and compacted local chromatin. More recently, there is increasing evidence that demonstrates the transcription activation role of PRC1. Moreover, studies on the higher-order chromatin structure have revealed a new function for PRC1 in mediating long-range interactions. This provides a different perspective regarding both the transcription activation and repression characteristics of PRC1. This review summarizes new advancements regarding the composition of mammalian PRC1 and accompanying explanations of how diverse PRC1-associated proteins participate in distinct transcription regulation mechanisms.

## 1. Introduction

The first Polycomb group (PcG) gene, Polycomb (*Pc*), was initially discovered in *Drosophila*, when *Pc* mutant larvae displayed dysregulated segmentation during development [1]. It was later shown that *Pc* functions as a transcriptional repressor of the *Hox* genes [2]. At least 16 PcG genes were subsequently identified by genetic screens in *Drosophila* with the criteria of an extra sex combs phenotype and the ectopic expression of *Hox* genes [3]. PcG genes are conserved in higher organisms and are critical players in many biological events, including embryonic development, stem cell self-renewal and differentiation, and carcinogenesis [4,5,6,7,8,9]. 

PcG gene products form at least two distinct types of protein complexes, Polycomb Repressive Complex 1 and 2 (PRC1 and PRC2), which regulate targeted gene transcription [7,10]. Biochemically, PRC1 is responsible for catalyzing the monoubiquitylation of histone H2A at lysine 119 (H2AK119ub1), while PRC2 is involved in mono-, di-, and tri-methylation of histone H3 at lysine 27 (H3K27me1/2/3) [11,12,13,14,15,16,17]. In mammals, the number of PcG genes are greatly expanded due to the creation of paralogs during multiple duplication events [18], although evidence suggests an earlier emergence of genetic repertoire for the diversity of PRC1 complexes during evolution [19]. This produces a far more diverse array of mammalian PRCs than their *Drosophila* counterparts and presents significant challenges when assessing their biological functions. 

Recent proteomic and biochemical analyses reveal that the core components of PRC1 include the E3 ubiquitin ligase RING1A/B and one of six Polycomb group RING finger (PCGF) proteins [20]. The exclusive association of RING1A/B with each PCGF gives rise to six groups of PRC1 complexes. In addition to these core components, numerous associated proteins also exist in different PRC1 complexes with each impacting PRC1 function in distinct ways [20,21,22]. Traditionally, PRC1 was considered to be a transcriptional repressor that catalyzes H2AK119ub1 and compacts chromatin [7,11,12,23,24,25]. Contradictory to its repressive role, emerging evidence indicates that PRC1 also activates transcription under certain circumstances [26,27,28,29,30,31,32]. Recent studies also have revealed a new function of PRC1 in the higher-order chromatin structure, in which it is involved in both transcription activation and repression [31,33,34,35,36,37,38]. We believe that the extensive diversity observed in the composition of PRC1 complexes is responsible for their ability to regulate transcriptional activities, as further discussed below.

## 2. Composition of PRC1 Complexes

In *Drosophila*, the core PRC1 complex contains Pc, Sex Combs Extra (Sce/dRing), Posterior Sex Combs (Psc), Polyhomeotic (Ph), and a sub-stoichiometric amount of Sex Comb on Midleg (Scm) [25,39]. A similar complex that contains various homologs of the core subunits of the *Drosophila* PRC1 complex was later purified from mammalian cells [18]. Since then, proteomic and biochemical analyses have shown that many additional polypeptides are associated with PRC1 [20,21,22]. It is likely that these different PcG homologs and associated factors form different complexes with each performing distinct functions. 

**Figure 1:**
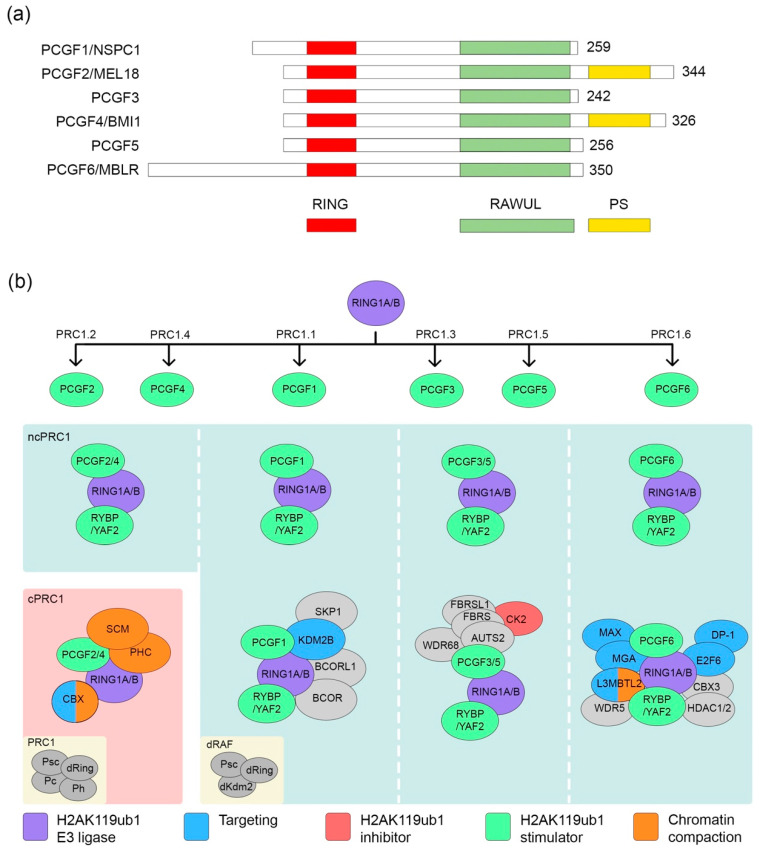
The domain architecture of Polycomb group RING finger (PCGF) proteins and the composition of Polycomb Repressive Complex 1 (PRC1) complexes. (**a**) PCGF proteins share highly conserved protein domains (RING and RING finger and WD40-associated ubiquitin-like (RAWUL)) and unique protein domains (PS) are only present in PCGF2/4; (**b**) The classification of PRC1 complexes. Based on PCGF proteins, PRC1 can be divided into six categories, PRC1.1–6. In the presence of RING1 and YY1 Binding Protein (RYBP)/YY1 Associated Factor 2 (YAF2) or CBX/PHC/SCM proteins, PRC1 can be classified as canonical PRC1 complexes (cPRC1) and non-canonical PRC1 complexes (ncPRC1). Inserts on the left bottom show the corresponding *Drosophila* complexes.

As a key step toward understanding the diverse functional impact of this important class of epigenetic modulators, studies conducted in the past decade have revealed the tremendous complexity of mammalian PRC1 complexes. One study that employed affinity purification followed by mass spectrometry analysis in HEK293 cells resulted in the identification of many novel PRC1 complexes in addition to confirming the existence of previously known complexes [20]. An interesting observation from this study was that mammalian PRC1 complexes fall into six groups based on their exclusive association with one of the six PCGF proteins (PCGF1/2/3/4/5/6; homologous to *Drosophila* Psc) (Figure 1). RING1A or RING1B, homologs of Sce/dRing, is a common component in each of these six groups, which are named PRC1.1-6, according to the associated PCGF. PRC1.2/4 contains various chromodomain proteins (CBX2/4/6/8) homologous to Pc, three Polyhomeotic Homologs (PHC1/2/3), and sub-stoichiometric amounts of Scm homologs (SCMH1, SCML1, and SCML2). Given the compositional similarity between the CBX/PHC/SCM-containing PRC1.2/4 and the initial *Drosophila* PRC1 complex, they are referred to as canonical PRC1 complexes (cPRC1) [20,40,41,42]. Other complexes including the RING1 and YY1 Binding Protein (RYBP) and its homolog YY1 Associated Factor 2 (YAF2) -containing PRC1.2/4 as well as PRC1.2/3/5/6 are considered non-canonical or variant PRC1 complexes (ncPRC1 or vPRC1) [20,40,41,42]. Following the initial characterization of mammalian PRC1 complexes, further research explored the biochemical architecture of these complexes and their functional impact. Interestingly, CBX7 was not found to be associated with PRC1.2/4 in the early study [20], even though it was present in later studies of mouse embryonic stem cells (ESCs) [22,41]. This is likely due to its differential expression among different cell types. In addition to subunits that are similar in *Drosophila* PRC1 counterparts, RYBP/YAF2 are present in PRC1.2/4 and have been shown to be mutually exclusive with CBX/PHC/SCM proteins [20,41,42,43]. Even though the classification of PRC1 complexes as PRC1.1–1.6 or cPRC1 versus ncPRC1 does not instantly distinguish their respective functions, this categorization is a useful stepping stone toward pinpointing the role(s) of individual complexes in transcription regulation.

### 2.1. PRC1.2/4

Among all PCGFs, PCGF2 and PCGF4 are most closely related to each other. In addition to the RING finger and WD40-associated ubiquitin-like (RAWUL) domain present within all PCGFs, these two PCGFs share a specific Proline Serine rich (PS) domain at the C-terminus (Figure 1a) [44,45,46,47]. Furthermore, the composition of PCGF2-containing PRC1.2 and PCGF4-containing PRC1.4 is identical [20]. This largely explains the functional redundancy between these two proteins and their associated complexes observed during development [48,49,50,51]. Both PRC1.2 and PRC1.4 contain cPRC1 and ncPRC1 complexes, unlike other groups that only have ncPRC1 complexes (Figure 1b) [20].

cPRC1.2/4 is characterized by the presence of CBX, PHC, and SCM proteins (Figure 1b), each of which confers distinct functions to the complex. Among eight known CBX proteins, five of them are present in cPRC1 and directly influence cPRC1 targeting [20,22,41]. The affinity of CBX proteins for H3K27me3 recruits the cPRC1.2/4 complexes to the PRC2 pre-occupied loci, which is essential for the hierarchical crosstalk between cPRC1 and PRC2 [52,53,54]. Despite a shared affinity for H3K27me3 among all PRC1-related CBX proteins, they also exhibit unique functions [54,55]. CBX2 but not CBX7 is characterized by a protein region with a disordered secondary structure and enriched basic amino acids and contributes to both the chromatin compaction and phase-separation both in vitro and in vivo [56,57,58]. In addition to the H3K27me3-based targeting mechanism, a recent study showed that the AT-hook of Cbx2 can bind to AT-rich major satellites DNA sequence [59]. Interestingly, CBX7 has been shown to bind non-coding RNA through chromodomains, which expands our understanding of PRC1 recruitment [60]. Likely, the dynamics and interplay of CBXs are key to ensuring the precise execution of their biological functions. For example, CBX6 and CBX7 are necessary for stem cell self-renewal, while CBX7 is replaced by CBX2/4/8 to ensure proper differentiation [22,61,62,63,64].

Three mammalian paralogs of PHC (PHC1/2/3) are associated with cPRC1 complexes [20]. PHC proteins mediate the formation of Polycomb bodies (PcG bodies), which are condensed chromatin structures enriched with PcG proteins and clustered with PcG repressed genes [65,66,67,68]. In both *Drosophila* and mammals, PHCs regulate PcG body formation via the sterile alpha motif (SAM) domain, which promotes the polymerization of PRC1 complexes [68,69]. SCM proteins also contain a SAM domain whose architecture is similar to that of PHCs. Through the SAM domains, these proteins are able to form polymers in vitro, which may be involved in mediating chromatin architecture [68,70,71].

Within PRC1.2/4, RYBP/YAF2 forms ncPRC1.2/4 with RING1A/B and PCGF2/4 in a mutually exclusive manner with CBX/PHC/SCM proteins (Figure 1b) [20]. Compared with cPRC1.2/4, recent genomic studies showed that ncPRC1.2/4 associates with a higher level of H2AK119ub1 in both *Drosophila* and mammals [20,40,72]. Similarly, ncPRC1.1/3/5/6 also exhibits higher H2AK119ub1 than cPRC1.2/4 [20,72,73]. RYBP may utilize multiple mechanisms to achieve this effect, which will be discussed later.

### 2.2. PRC1.1

PRC1.1 in mammals contains other components besides RING1A/B and PCGF1, including Lysine Demethylase 2B (KDM2B), BCL6 Corepressor (BCOR) and its homolog BCL6 Corepressor Like 1 (BCORL1), S-phase kinase-associated protein 1 (SKP1), ubiquitin-specific-processing protease 7 (USP7), and RYBP/YAF2 (Figure 1b) [20]. A KDM2B-containing complex named dRing-associated factors (dRAF) complex was previously identified from *Drosophila* and contains three components, dRing, Psc, and dKdm2 (Figure 1b) [74]. Known as a H3K36me2 demethylase, KDM2B helps PRC1.1 target to chromatin, surprisingly, independent of its histone demethylation activity [75,76,77,78]. In the process of investigating how PcG complexes recognize their target loci, genomic analysis showed that CpG islands (CGI) are tightly associated with the genomic occupancy of PRC1/PRC2 [79]. Mechanistically, the CxxC-zinc finger domain of KDM2B recognizes unmethylated CpG islands and drives the complex toward target loci [75,76,77,80]. Upon recruitment by Kdm2b, PRC1.1 binds to its target genes and deposits H2AK119ub1, which then represses transcription in mouse ESC [80]. Surprisingly, Wang and colleagues have shown that the deletion of KDM2B in human ESCs did not severely affect the recruitment of PRC1 to critical differentiation genes; instead, another PRC1.1 component, BCOR, was necessary and sufficient for both PRC1 targeting and repression [81]. BCOR was previously known to link PRC1.1 and KDM2B/SKP1 by forming a heterodimer with PCGF1, along with its homolog BCORL1 [82,83]. The observation of cell type-specific functions of KDM2B and BCOR/BCORL1 in PRC1.1 recruitment may indicate distinct targeting mechanisms in different species.

### 2.3. PRC1.6

PRC1.6 contains several DNA- or chromatin-associating factors that may contribute to its recruitment to target loci (Figure 1b) [84,85,86,87,88]. The presence of MAX gene-associated protein (MGA) and E2F Transcription Factor 6 (E2F6) in PRC1.6 complexes establishes a foundation for sequence-based PRC1.6 recruitment [84,87]. MGA, along with MYC-associated factor X (MAX), recognizes and binds to a DNA motif E-box [89]. Meanwhile, E2F6 forms a heterodimer with DP-1/2 to bind to an E2F recognition sequence [84,89,90]. While the collaboration between these two components mediates the DNA-based recruitment, another core component of PRC1.6, L3MBTL2, has the potential to regulate the chromatin-based recruitment of PRC1.6 [85,86,88,91]. It has been shown that four MBT domains on L3MBTL2 can bind to mono- and di-methylated histone H3 and H4 in vitro, and later studies demonstrated that the first two MBT repeats alone are sufficient for PRC1.6 chromatin targeting [86,88,91]. Together, these studies illustrate the complexity of PRC1.6 recruitment and the degree to which it is affected by the orchestration of DNA sequence and histone modifications. 

Similar to other groups of PRC1 complexes, PRC1.6 is capable of depositing H2AK119ub1 at target loci [84,85,92]. Other mechanisms may contribute to its suppressor function as well. It has been shown that L3MBTL2 can compact chromatin in vitro independent of histone modifications [85]. Moreover, histone deacetylase HDAC1/2 (components of PRC1.6) confers PRC1.6 histone deacetylation activity [92]. Furthermore, histone methyltransferase G9A also assists PRC1.6 with depositing H3K9me1/2 [92]. It is worth mentioning that WDR5 and CBX3, which are factors associated with other chromatin-modifying complexes as shown previously [93,94], are also present in PRC1.6 [20]. It will be interesting to see how future studies describe the influence of WDR5 and CBX3 on PRC1.6 function and whether they provide a mechanism for orchestrating various epigenetic pathways.

### 2.4. PRC1.3/5

PCGF3 and PCGF5 share a group of interactors, including AUTS2, FBRS, FBRSL1, WDR68, and CK2 (Figure 1b) [20]. Although it is not clear how PRC1.3/5 is recruited to chromatin through these components, studies suggest that several DNA-binding transcription factors may be involved in its targeting. It has been shown that ChIP-seq targets are largely overlapped between PCGF3 and Upstream stimulatory Factor 1 (USF1), and knockdowns of both USF1 and USF2 lead to the reduction in PCGF3 chromatin association [73]. Testis Expressed 10 (Tex10) also has been found to physically interact with PCGF3/5, and this interaction is required for its recruitment to selective targets [27]. In addition to recruitment by transcription factors, PRC1.3/5 also can be targeted to the X chromosome by long non-coding RNA Xist and the Xist-interacting protein, Heterogeneous Nuclear Ribonucleoprotein K (hnRNPK), to establish polycomb-mediated X chromosome inactivation [95,96]. Within PRC1.3/5, PCGF5 and presumably PCGF3 directly interact with AUTS2. AUTS2 makes direct contact with CK2 and P300, which are two factors that contribute to the transcriptional activation by PRC1.3/5 [26,27,97]. FBRS and FBRSL1 are homologs of AUTS2, but their roles in PRC1.3/5 remain unknown. Recently, WDR68 has been found to be critical for PRC1.3/5-mediated transcriptional activation and neuronal differentiation from mESCs [28]. 

## 3. PRC1 in Transcription Regulation

**Figure 2:**
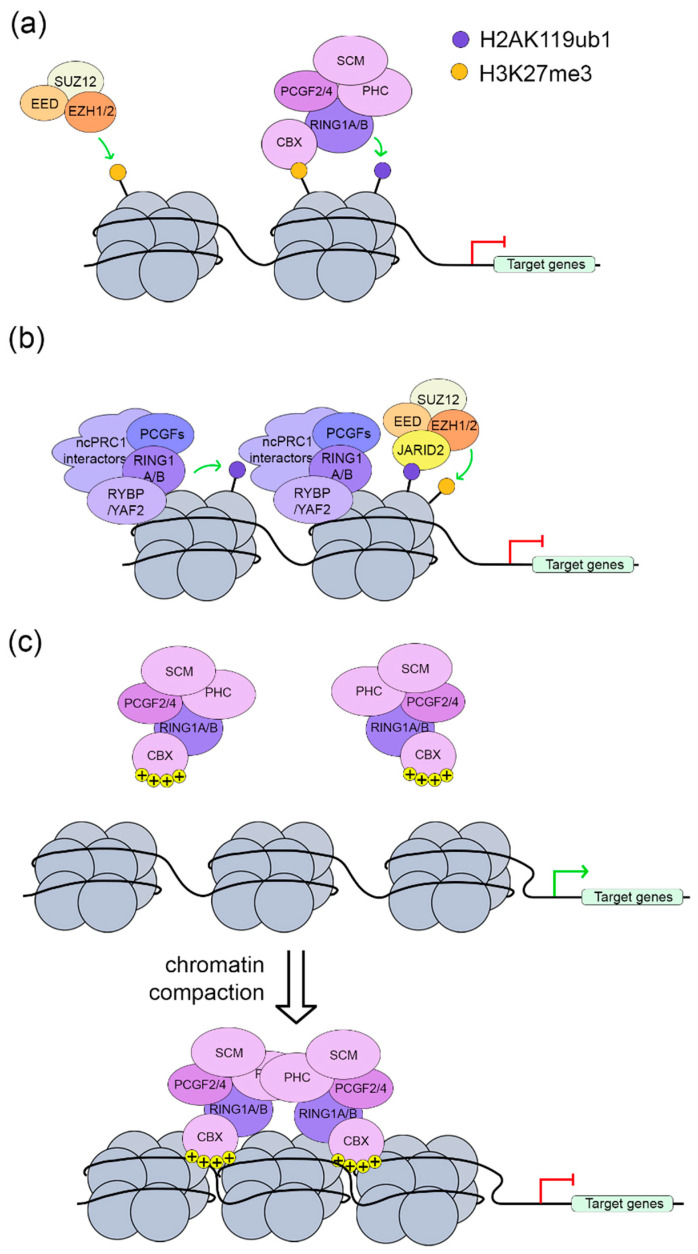
PRC1-mediated transcription repression. (**a**) Crosstalk between cPRC1 and PRC2. PRC2 deposits tri-methylation of histone H3 at lysine 27 (H3K27me3) to genomic loci. Then, CBX proteins drive cPRC1 to PRC2 pre-occupied loci and deposit monoubiquitylation of histone H2A at lysine 119 H2AK119ub1; (**b**) Crosstalk between ncPRC1 and PRC2. ncPRC1 approaches the genomic loci first and catalyzes H2AK119ub1; PRC2 recognizes and occupies the same region through JARID; (**c**) cPRC1 complexes mediate chromatin compaction to repress target genes, which is mediated by the interaction between positively charged region of CBX proteins and nucleosomes, and the self-polymerization of PHC proteins.

Mounting evidence has shown that PRC1 complexes repress gene transcription through chromatin modifications [7,10]. Two mechanisms have been suggested to account for the mediation of this gene silencing: H2AK119ub1 and local chromatin compaction (Figure 2) [11,12,23]. Recent mechanistic studies have offered new advances in our understanding of how these activities contribute to transcriptional repression. Surprisingly, several studies also showed an activating role of PRC1 during gene transcription [26,27,28,29,30,31,32]. Despite the challenges in elucidating the roles of the vast number of PRC1-associated factors in transcriptional activation, we are beginning to uncover mechanistic insights regarding this PRC1 paradox. Recent advances in 3D chromatin architecture also have revealed the involvement of PRC1 in long-range chromatin interaction, which suggests yet another regulatory function of PRC1 during transcription [33,34,35,36,37,38].

### 3.1. PRC1 in Transcription Repression

As the common component of all mammalian PRC1 complexes, E3 ubiquitin ligase RING1A/B encodes the catalytic activity for H2AK119ub1 deposition [11,12]; this provides a general mechanism for PRC1-mediated transcriptional repression. Genome-wide studies have shown a strong correlation between the occupancy of H2AK119ub1 and gene repression [73,98,99,100]. The counteracting effect of H2AK119ub1 on RNA polymerase II supports the repressive function of H2AK119ub1 and provides a mechanistic explanation of how the PRC1 complex regulates gene expression through histone modification [98,101]. As a multicomponent complex, other PRC1-associated factors also are involved in the regulation of RING1A/B ubiquitination activity. PCGF proteins, working through the RING domain, bind to RING1A/B and enhance its enzymatic activity [102,103]. Noticeably, compared with ncPRC1-containing heterodimers (Pcgf1/3/5/6 with Ring1b), cPRC1 heterodimers (Ring1b with Pcgf2/4) display lower E3 ligase activity [102]. With the overall similar structure between the cPRC1–RING1B and ncPRC1–RING1B heterodimers, the significant difference in enzymatic activity may result from the salt bridge in the E2–PCGF2/4 interface, which limits the efficiency of ubiquitin transfer [102]. In addition to PCGF proteins, in vitro ubiquitination assays have revealed that RYBP/YAF2 can also robustly promote RING1A/B activity [20,102]. In fact, RYBP enhances the E3 ligase activity of all six PCGF–RING1B dimers [72,102]. Although this is in agreement with the observation that the occupancy of RYBP correlates to higher H2AK119ub1 levels and gene repression, we cannot rule out other mechanisms [20,40,72]. It has recently been found that RYBP is required in the propagation of H2AK119ub1 across the cell cycle dependent on its binding to this modification [104]. It will be interesting to be seen whether this interaction may play any role in affecting global levels of H2AK119ub1. Additionally, USP7, a PRC1.1-associated protein and a deubiquitinase, was found to also associate with PRC1.4 [105]. This interaction with USP7 prevents the self-ubiquitination of PRC1.4 components including RING1B and PCGF4, leading to the stabilization of the PRC1.4 complex and hence the H2AK119ub1 level. Clearly, H2AK119ub1 plays an important role in gene repression by PRC1 and further understanding how its ubiquitination activity is precisely regulated by associated PCGFs; RYBP/YAF2 and other factors will likely unlock new mechanistic insights into PRC1-mediated epigenetic regulation. 

The PRC2 complex is one of the factors that intimately collaborates with PRC1 to target, establish, and maintain the PcG target gene repression (Figure 2) [52,53]. This collaboration has initially been regarded as hierarchical; PRC2 approaches target loci and deposits H3K27me3, which is followed by the recruitment of PRC1 [52,106]. Since CBX proteins recognize H3K27me3, they drive cPRC1 to PRC2 pre-occupied loci (Figure 2a) [14,52,106]. The essential role of CBX proteins indicates that this model can only apply to the recruitment of cPRC1 but not ncPRC1. Indeed, RING1B can occupy loci that lack H3K27me3, which demonstrates that PRC1 can be recruited to the chromatin in a PRC2-independent manner (Figure 2b) [20,42,107]. Recent studies tackling the PRC1 and PRC2 interplay revealed surprising findings contradictory to our previous knowledge [40,108,109]. Using an artificial cellular targeting system, it has been shown that ncPRC1-mediated H2AK119ub1 recruits PRC2 [40]. Later studies showed that this mechanism is important for X-inactivation, as prior PRC1.3/5 binding leads to PRC2 recruitment [95,96]. Similar to the essential role of CBX proteins in cPRC1–PRC2 crosstalk, Jumonji and AT-Rich Interaction Domain Containing 2 (JARID2), a PRC2 component, is able to bind to H2Aub, thus illuminating the molecular link of how PRC2 is recruited by ncPRC1 enzymatic activity [108].

PRC1 also can repress gene expression through H2AK119ub1-independent chromatin compaction (Figure 2c) [23,24,25,34]. Initial in vitro assays with nucleosomal arrays revealed that the *Drosophila* PRC1 complex can compact chromatin, independent of histone modifications [23,25]. In fact, the Psc subunit alone is sufficient to conduct such activity [23]. Later work in mouse ESCs demonstrated this observation in vivo using a catalytically inactive mutant Ring1b [24]. Despite the observation of unchanged H2AK119ub1 level at the *Hox* loci, this mutant was able to rescue the loss of chromatin compaction as well as the derepression of *Hox* genes caused by Ring1b deletion in mouse ES cells [24,34,110]. Even though Psc plays a critical role in *Drosophila*, CBX proteins carry out the compaction function in mammals. CXB2 and Psc share a disordered region enriched with highly positively charged amino acids that is distinct from other mammalian PCGFs, and this region is crucial to induce chromatin compaction in vitro [56,57]. Interestingly, CBX7 does not have this domain and hence lacks compaction ability [56,57]. This indicates non-compensatory functional diversity even among closely related CBX-containing cPRC1 complexes. Importantly, in mouse models carrying Cbx2 with a mutated nucleosome compaction region, Lau et al. demonstrated that the compaction function is necessary for normal PRC1-mediated *Hox* gene expression and axial patterning [111]. Although not required for nucleosomal compaction in vitro [23], PHC and SCM proteins may contribute to chromatin compaction in vivo through SAM-mediated self-polymerization [68,69]. Chromatin compaction activity is not solely attributed to cPRC, as L3MBTL2, a PRC1.6 component, also can compact nucleosomes [85], yet the underlying mechanism and its relevance to PRC1.6-mediated transcriptional repression remain unclear. 

### 3.2. PRC1 in Transcription Activation

Traditionally, PRC1 is known as a transcription repressor. However, an opposite role of PRC1 as a transcription activator has been revealed recently [26,27,28,29,32,36]. Using a murine megakaryoblastic cell line, Yu et al. observed that Runx1 and Cbfβ share substantial target loci with Ring1b and more interestingly, a knockdown of Ring1b leads to both the up- and down-regulation of target genes [30]. This indicates a role for PRC1 in both transcriptional repression and activation. Subsequent studies in various cell types revealed the involvement of multiple PRC1 components in active transcription, including Pcgf1, Cbx8, and Ring1b [32,112,113,114]. However, these studies have not provided the molecular mechanisms underlying PRC1-mediated transcriptional activation. 

**Figure 3:**
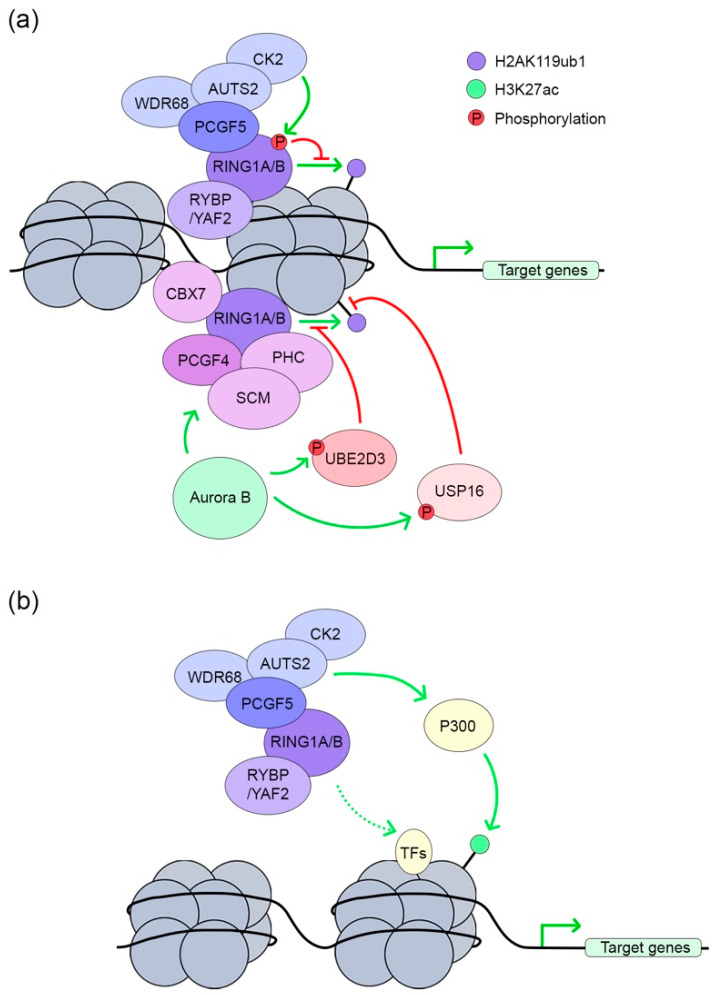
PRC1-mediated transcription activation. (**a**) Some PRC1-associated proteins directly or indirectly neutralize the H2AK119ub1 enzymatic activity of PRC1; (**b**) AUTS2-containing PRC1.3/5 is recruited by transcription factors and sequentially drives P300 to the same loci for acetylation of histone H3 at lysine 27 (H3K27ac).

As described above, the E3 activity of Ring1b is enhanced by several other PRC1 components to achieve optimal H2A mono-ubiquitination and hence gene repression [20,53,72,102,103,114]. Recently, factors have been identified that inhibit Ring1b-mediated H2A mono-ubiquitination, providing mechanistic insights into PRC1-mediated transcriptional activation. Biochemical characterization of the PCGF5-containing PRC1.5 complex has led to the identification of additional components including AUTS2 and CK2 [26]. Interestingly, in vitro reporter assays suggest a role for PRC1.5 in transcriptional activation [26]. In contrast to canonical PRC1 complexes, PRC1.5 tend to localize to genomic loci lacking H2AK119ub1, indicating compromised mono-ubiquitination activity. In fact, the stable association of CK2 leads to the neutralization of RING1B enzymatic activity by phosphorylating RING1B at serine 168 (Figure 3a, top) [26]. Another study has found an alternative mechanism responsible for the inhibition of H2AK119ub1 (Figure 3a, bottom) [29]. In mouse quiescent lymphocytes, PRC1.4 colocalizes with Aurora B kinase at active promoters [29]. In contrast to the Ck2-mediated direct phosphorylation of Ring1b, Aurora B kinase inhibits H2A mono-ubiquitination by phosphorylating and deactivating the E2 enzyme Ube2d3; it also promotes H2A de-ubiquitination by phosphorylating and enhancing the enzymatic activity of a deubiquitinase, Usp16. 

In addition to the inhibition of the intrinsic inhibitory activity of PRC1, other mechanisms have been suggested to explain its role in transcriptional activation. The presence of AUTS2 in the PRC1.5 complex not only recruits CK2 but interacts with P300, which is a histone acetyltransferase and transcriptional co-activator [26,27]. The knockdown or pharmacological inhibition of P300 compromises the PRC1.5-mediated induction of a luciferase reporter [26], suggesting an important role for P300 in the transcriptional activity of this complex. The involvement of P300 with PRC1.5 and PRC1.3, which share the same complex composition, in activating gene transcription has been suggested in later studies (Figure 3b) [27,97,115,116]. Although Auts2 was not detected in the PRC1.3/5 isolated from mouse ES cells, which is likely due to its low expression in this cell type, Tex10 has been found to associate with PRC1.3/5 and may play a role in recruiting P300 [27].

### 3.3. PRC1 in Chromatin Architecture

The eukaryotic genome is properly folded in the nucleus. Chromosomes are organized on a large scale into mutually exclusive regions known as A/B compartments in which the A compartment is enriched with transcriptionally active genes and the B compartment corresponds to repressive chromatin [117,118]. At megabase scales, the genome segregates into self-interacting regions, which are known as topologically associating domains (TADs) [119,120]. Within TADs, two distal DNA sites are brought spatially close to each other by bending the chromatin fibers, thereby forming chromatin loops. While TADs are conserved among cell types and species, loops are highly dynamic, which allows for the precise regulation of genes on the loop anchors [119,121]. The chromatin structure is shaped by various proteins with CTCF and a cohesin complex bearing primary responsibility for the majority of TAD and looping formation [118,119,120,122]. However, studies in *Drosophila* showed a strong correlation between loop anchors and PRC1 chromatin occupancy, which suggested a potential contribution by the PRC1 complexes to loop formation [33,34]. Furthermore, the dynamic of Ring1b binding affects the PRC1-mediated looping formation and subsequently regulates target gene expression during mouse ESC differentiation [123].

**Figure 4:**
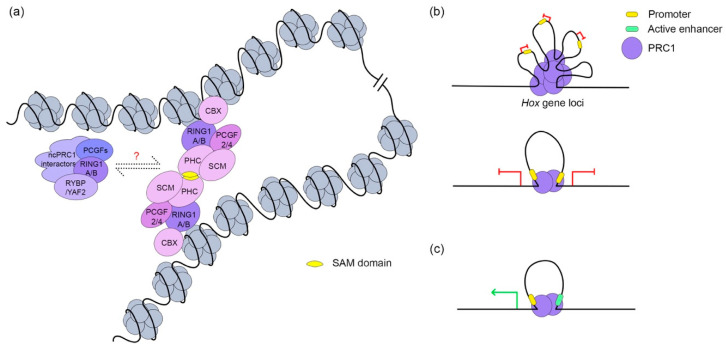
PRC1-mediated looping formation. (**a**) PRC1-mediated looping formation depends on the polymerization of PHC proteins’ sterile alpha motif (SAM) domain. It is unclear if ncPRC1 also is involved in long-range chromatin architecture regulation; (**b**) PRC1-mediated loops work in two patterns to repress anchored genes, either by chromatin compaction or co-occupied two promoters; (**c**) PRC1-mediated loop establishes contact between the promoter and active enhancer to activate anchored gene expression.

By analyzing the classic PcG target loci, it has been shown that PRC1 complexes brings the distal ends of *Hox* gene loci spatially close to each other to achieve transcription co-repression (Figure 4) [24,68]. Through promoter capture HiC analysis, the PRC1 complex has been shown to organize high-order chromatin architecture [38]. A possible model for PRC1-mediated looping is the polymerization of PcG complexes, presumably the cPRC1.2/4, which brings Phc proteins into the spotlight. As mentioned above, the C-terminus of Phc proteins contains a highly conserved SAM domain, which is characterized by head to tail self-polymerization in vitro (Figure 4a) [70,71]. To test the necessity of Phc proteins in the looping formation, Kundu and colleagues generated Phc1 knockout mouse ESC cells [34]. The loss of Phc1 proteins led to a sharp decrease in the chromatin occupancy of canonical PRC1 components together with the disruption of PRC1-mediated chromatin loops [34]. This observation agrees with those of an earlier study in which a dominant negative form of a mutant Phc2 SAM domain, which was incapable of polymerization, disrupted the PRC1-mediated loop formation and the repression of genes targeted by chromatin condensation (Figure 4b) [68,71]. Using a similar method, the necessity of the presence of a SAM domain in the PRC1-mediated repressive chromatin structure was also confirmed in *Drosophila* [69]. These studies clearly indicate that PRC1 complexes are essential for chromatin looping, but additional research is needed to further elucidate the underlying molecular mechanisms.

Since PRC1 exhibits a dual-directional regulation of transcription, PRC1-mediated loops also can mediate active transcription in addition to repression. In a study aimed at understanding how PRC1 regulates the tissue-specific expression of *Meis2*, Ring1b was found to bind to both the promoter of the *Meis2* gene and a tissue-specific enhancer in an early developing midbrain where *Meis2* is highly expressed [31]. The occupancy of PRC1 at both the promoter and tissue-specific active enhancer establishes a new loop to achieve gene activation (Figure 4c) [31]. This is of great interest because in other regions of developing brains with a low expression of *Meis2*, Ring1b is found to simultaneously bind to the promoter and the 3′ end of the *Meis2* gene to form a loop leading to the tissue-specific transcriptional repression of *Meis2* [31]. This study suggests that the genomic elements of the PRC1-mediated loop’s anchors determine its impact on gene transcription. The activation function of a promoter-enhancer PRC1-mediated looping is further confirmed by the genome screen of *Drosophila* eye-antennal imaging discs (Figure 4c) [36]. This recent study revealed that promoter-enhancer loops is a common method used by PRC1 to fine-tune gene expression. In fact, more than half of PRC1-mediated loops are between promoters and enhancers [36]. This transcriptional activation mechanism mediated by PRC1-loops is likely present in higher organisms, as a study showed that during the neural differentiation of mouse ESCs, PRC1 dynamically establishes enhancer-promoter loops to activate differentiation-related genes [36,123].

## 4. Future Directors

Decades of scientific effort have greatly expanded our understandings of the composition and biological function of the PRC1 complex. With a combination of biochemical and proteomic analyses, numerous associated proteins have been identified to date. Our obtained knowledge in the complexity of PRC1 composition will likely facilitate the future dissection of various activities and functions of PRC1 complexes. Combining recent technological development in gene editing, stem cell biology, and single cell transcriptome analysis, we envision a much deeper understanding of how individual PRC1 complexes coordinate to determine the cell type-specific epigenome and transcriptome through the orchestration of various mechanistic actions. In the past decade, with the rapid development of sequencing technologies, we have begun to uncover the chromatin occupancy of PRC1 and the influence of PRC1 on chromatin 3D structures. It has been shown that PRC1 functionally interacts with CTCF and cohesion complex to control the long-range chromatin interactions [35,124]. However, much work is needed to understand the molecular transactions of this interplay. Going forward, these studies hold great promise for further scrutinizing PRC1’s specific function(s) and mechanism(s) of regulating transcription. 

## Abbreviations

BCOR	BCL6 Corepressor
BCORL1	BCL6 Corepressor Like 1
CBX	Chromodomain protein
CGI	CpG island
dRAF	dRing-associated factor
E2F6	E2F transcription Factor 6
ESC	Embryonic stem cell
H2AK119ub1	Monoubiquitylation of histone H2A at lysine 119
H3K27ac	acetylation of histone H3 at lysine 27
H3K27me	Methylation of histone H3 at lysine 27
hnRNPK	Heterogeneous Nuclear Ribonucleoprotein K
JARID2	Jumonji And AT-Rich Interaction Domain Containing
KDM2B	Lysine Demethylase 2B
MAX	MYC-associated factor X
MGA	MAX gene-associated protein
Pc	Polycomb
PcG	Polycomb group
PCGF	Polycomb group RINGring finger
Ph	Polyhomeotic
PHC	Ph homolog
PRC	Polycomb Repressive Complex
Psc	Posterior Sex Comb
RAWUL	RING finger and WD40-associated ubiquitin-like
RYBP	RING1 and YY1 Binding Protein
SAM	Sterile alpha motif
Sce	Sex Combs Extra
Scm	Sex Comb on Midleg
SKP1	S-phase kinase-associated protein 1
TAD	Topologically associating domains
USP7	Ubiquitin-specific-processing protease 7
YAF2	YY1 Associated Factor 2
